# The Early Introduction of Complementary (Solid) Foods: A Prospective Cohort Study of Infants in Chengdu, China

**DOI:** 10.3390/nu11040760

**Published:** 2019-04-01

**Authors:** Chuan Yu, Colin W. Binns, Andy H. Lee

**Affiliations:** 1 Department of Health-Related Social and Behavioural Science, West China School of Public Health, Sichuan University, Chengdu 610041, China; chuan.yu@scu.edu.cn; 2 School of Public Health, Curtin University, GPO Box U1987, 6845 Perth, Australia; Andy.Lee@curtin.edu.au

**Keywords:** complementary foods, child health, China, infants, cohort study

## Abstract

The objective of this study was to document the types of foods introduced to infants before six months of age and identify factors associated with their early introduction. A prospective cohort study of infant feeding for the first six months after birth was undertaken in the city of Chengdu, PR China. The participants were 845 mothers who delivered their infants in hospitals in Chengdu. Mothers were interviewed within 15 days of giving birth and were followed up with for six months. The outcome measures were the introduction of complementary foods to infants within four and six months postpartum. Complementary foods are defined as any food, whether manufactured or locally prepared, used as a complement to breast milk or infant formula. In this study the emphasis was on solids and not liquid foods. More than 94% of the infants were given complementary foods (semi-solid or solid foods) before the age of six months and 10% by four months. The most commonly introduced food was infant cereal, which was given to three quarters of the infants by six months. Multivariate analysis showed that maternal education level was a significant factor affecting the introduction of complementary foods before four months, adjusted odds ratio 2.983 (1.232–7.219), with the more educated mothers introducing complementary foods earlier. More antenatal and postnatal health promotion efforts are required to highlight the benefits of introducing solid foods later than is the current practice in Chengdu, at or close to six months of age. Further education is also required for training health professionals including pediatricians, midwives, and community health staff.

## 1. Introduction

Human infants require complementary foods at about six months of age to provide adequate nutrition during the transition from breast milk to a family diet. This is unique among mammalian species and the period of complementary feeding typically covers the period from six to 24 months, even though mothers may well choose to continue breastfeeding beyond this period [1]. Breastfeeding brings many benefits to the health and development of infants including long term benefits into adult life [2,3,4,5]. Despite its many nutritional attributes, beyond six months of age breast milk requires the addition of complementary foods to provide adequate nutrition for infants [1]. 

The introduction of complementary (solid) foods at around six months is important because early or late introduction can cause health difficulties for the infant. The early introduction of solids increases the risk of diarrheal disease, food allergies, and probably increases the rate of being overweight in infancy and childhood [6,7]. It is estimated that worldwide there are 41 million children under five years old who are obese or overweight, and recent reviews have strengthened the reported association between the early introduction of solids, particularly introduction before the age of four months, and higher protein foods and obesity [8,9,10,11]. The gastrointestinal microbiome is influenced by early infant feeding practices, including the early introduction of complementary foods. It is important for healthy infant development. Exclusive breastfeeding is associated with development of an optimal microbiome that has the better long-term health outcomes. Favorable gut colonization through continued breastfeeding beyond six months of age may promote tolerance, as well as provide protective factors when complementary feeding is initiated [12]. The late introduction of nutritious complementary foods (after six months of age) predisposes infants to micronutrient deficiencies, especially in lower income countries [13,14].

In Asian populations complementary foods are often introduced earlier than the recommended ages with a median age of introduction being four months in the region [2,15,16,17,18]. There are no recently published studies on the introduction of solid foods and the types of foods given by parents from Western China.

The aim of this study was to document the types of foods introduced to infants before six months of age in Chengdu, Western China, and to identify factors associated with their early introduction. 

## 2. Materials and Methods

For this prospective cohort study, a sample of 845 mothers, who delivered their infants in one hospital for women and children and five community health centers in the City of Chengdu (population 6.5 million) from April 2010 to January 2012, was selected. The eligibility criteria for inclusion were mothers who had a singleton birth in Chengdu during the recruitment period and who confirmed their babies would receive continuing child health care services from one of the above institutions for at least six months after birth. Exclusion criteria were the presence of major congenital abnormalities, births of less than 34 weeks gestation, and infants who had stayed in the neonatal intensive care unit for four days or longer after birth. Further details have been previously published [19]. In summary, we recruited mothers delivering their infants in the Sichuan Provincial Hospital for Women and Children and the five community health centers in the City of Chengdu that were associated with them. The great majority of mothers were primiparous and stayed in hospital for about 4 days. The in-hospital delivery rate in Chengdu was 100%. It was planned to recruit the sample continuously and unselectively until the required sample size was reached. However, on occasion there were too many deliveries to interview adequately and at these times the selection of mothers to be interviewed was made using random numbers. The hospital and health centers were visited three times a week and all the mothers present in the hospital were visited. Mothers who met the selection criteria were invited by the researchers to participate in the study and were interviewed after informed consent was given.

This study was part of a larger project on infant feeding in Chengdu. Sample size calculations were based on an estimated rate of ‘any breastfeeding’ at six months of 65% based on our previous studies in China and then allowed for a 10% dropout rate. We ended up with 760 participants, which was very close to the 10% dropout estimate. A sample size of 750 was estimated to give confidence intervals of +3%. This sample size would also have sufficient power to investigate the study objective on complementary foods. To account for an estimated dropout rate of 10% we estimated a sample of 825 would be required. A total of 890 mothers who met our inclusion criteria were contacted before discharge, we recruited 845, and at six months had a total of 760 remained, a dropout rate of 10.1%.

All participants were interviewed face to face after the birth of their infant and were followed up with through telephone interviews at one, three, and six months using structured questionnaires. The baseline and follow-up questionnaires used in this study were based on the questionnaires used in previous studies in Australia [20,21], Vietnam [22], the Xinjiang Uygur Autonomous Region, and Zhejiang Province of China [23,24]. The baseline questionnaire included the mother and her family’s socio-demographic information, baby’s information, breastfeeding information, and pregnancy and delivery information. The follow-up questionnaire included sections focusing on breastfeeding practices and feeding behaviors. The health status of infant and mother, the introduction of complementary food, and the use of health services were included in the follow-up questionnaire. The introduction of solid foods was defined as “regular use”. 

Complementary foods are defined as any food, whether manufactured or locally prepared, used as a complement to breast milk or infant formula [25]. Nutritional or dietary supplements, such as vitamin preparations, were not included in the definition. Solid foods should be nutritious, culturally appropriate, and acceptable in taste without added sugar, honey, or salt, starting with pureed foods and changing to normal texture by about 12 months [26]. 

This study was approved by the Human Research Ethics Committees of Curtin University (Approval number HR168/2009) and the participating hospital in China. The mothers were informed about the purpose of the study and their rights by an information letter at recruitment. Their participation was entirely voluntary and they could withdraw from the study at any time without prejudice. All mothers were reassured that their participation or nonparticipation in this study would not influence their baby’s health care in any way. 

All data were cleaned and screened for outliers, coding errors, and missing values. When errors were detected, which were very few in number, they were corrected from the original questionnaire, from medical records, or by telephone calls to the respondent. The data was subsequently analyzed using SPSS version 18.0. Descriptive and univariate analyses were used to describe the socio-demographic characteristics and other variables. Univariate and multivariate logistic regression models were conducted to determine the influencing factors associated with the introduction of solid foods before four months and six months of age. Potential variables considered were based on the available information and plausible factors from the literature. The variables included were maternal age, maternal education level, maternal occupation, family monthly income, gender, birth weight, attendance at antenatal classes, delivery method, parity, infant admission to special care nursery, the time mother went back to work, paternal smoking, feeding method at baseline, feeding method at one-month postpartum, breastfeeding problems of mothers by one month postpartum, and viewing advertisements related to infant feeding and family support.

## 3. Results

A total of 845 out of 890 mothers recruited at baseline met our eligibility criteria and agreed to participate in the study, a response rate of 95%. There were no significant differences in demographic characteristics between the participants and non-participants. During the six month follow up period 85 mothers left the study and 760 (89.9%) participants were included in the final analysis. Most of the mothers who left the study did so because they had moved out of the study area. The mean age of participants was 28.2 years. Chengdu is a centre of technology, and 67% of mothers had completed university education with 76% having a family monthly income greater than 5000 RMB. More than 80% of mothers were employed and approximately half returned to work within six months postpartum. Caesarean deliveries accounted for 65% of the total, consistent with the national average of 64.1% in Chinese cities and reflected the trend towards increasing operative delivery [27,28,29]. 

The demographic characteristics of the mothers at baseline (*n* = 845) and those who remained in the study at six months (*n* = 760) are shown in Table 1. The gender ratio (M/F) at birth was 1.12, similar to previous demographic reports [30]. Most of the mothers (88.9%) were primiparous reflecting the population policy of the time. 

The timing of the introduction of solid foods was associated with the years of education of mothers as shown in Table 2. Only one mother had received less than 10 years of education.

Almost all infants (94%) had been given complementary foods before six months. Almost all (90%) infants had been given water before four months. The complementary foods were classified into 11 categories in Table 3. The most frequently introduced complementary solid food before six months of age was infant cereal, given to more than 75% of infants. About 9% of mothers gave their babies fruit juice or vegetable juice before six months. 

While no infants were given solid food before one month, 6.6% had been introduced by four months. The median age of introducing solid foods was five months. Eight infants had been introduced to some solid foods before three months, and 50 infants by four months. The most common period for introducing solid foods was between four and six months. Only 49 mothers had not given any solid food to their infants by six months.

Solid foods first introduced to infants were rice porridge, infant cereal, noodles, protein foods, chicken liver, fruit, and vegetable paste. The protein foods included egg and meat. The vegetable and fruit pastes referred to homemade paste prepared from fresh vegetables or fruits. Among these seven food groups, only infant cereal and protein foods were introduced before four months. Infant cereal was the most frequently introduced solid food before six months of age. The earliest introduction of infant cereal was only six weeks after birth and about 6% of mothers gave their babies infant cereal before four months. Only 21.6% of mothers did not introduced infant cereal by six months postpartum (see Table 3). Though no infants were fed fruit paste or vegetable paste before four months, about half of them were given fruit paste (49.7%) and vegetable paste (45.9%) during the four to six months period. Few mothers gave rice porridge or noodles between four and six months (5.5% and 1.3%, respectively), which had been replaced by packets of processed cereal. 

The factors found associated with the early introduction of solid food before four months are shown in Table 4. Mothers who had completed 12 years of education were more likely to introduce solid food to their babies before four months than others with a lower educational level (odds ratio (OR) = 2.341). Mothers who were employed and were local residents in Chengdu were more likely to introduce solid foods before four months; OR = 8.315 and 2.096, respectively. The timing of return to work (before six months) was also associated with the introduction of solid food before four months, OR = 2.173. Multivariate logistic regression analysis further confirmed the significant association with ‘maternal education level’, with an increased likelihood of introduction by more educated mothers (OR = 2.983, 95% confidence interval (CI) 1.232–7.219), but not the other three factors identified in the univariate analysis. The results related to the introduction of solid food before six months are not shown as almost all infants (94%) had commenced solid foods by this age and there were no significant influencing factors.

## 4. Discussion

The early introduction of solid foods is a common practice in many cultures. Some mothers believe that it can reduce sleep interruptions by increasing the time between overnight feeds, and can increase the baby’s growth rate, the latter being viewed as beneficial in some cultures [18]. However, the early introduction of solid foods may also increase infant morbidity and mortality, though it is difficult to separate this effect from the decrease in breastfeeding and the dysbiosis of the microbiome. In many countries foods and water supplies are likely to be contaminated with bacteria, resulting in gastrointestinal infection [31,32,33]. 

The most common foods introduced to infants under six months of age in Chengdu were cereal products. In the cities in China this is invariably a packaged fortified product, similar to infant cereal products in western countries. Other common first solid foods introduced to infants were rice porridge, noodles, fruit paste, vegetable paste, protein food, and chicken liver. This pattern of infant foods was similar to the types of solid food introduced to infants in other cities of China [34,35,36]. In previous decades rice porridge would have been very common, but in this study it was only fed to about 5% of infants before six months. A dietary survey of five Chinese cities in 2012 (*n* = 750) found that all infants had received solids by six to eight months, with manufactured infant foods (cereals) being consumed by 36% of infants within the past 24 h [36]. The most commonly consumed complementary foods varied between different studies, different cities, and provinces. In Beijing, for example, the food most frequently introduced to infants was vegetables [34]. In a national survey of nine Chinese cities, the most common solid food given to infants was eggs [35]. 

Most infant feeding guidelines, including in China, recommend the introduction of complementary food at six months after birth [37,38]. Current recommendations are to introduce nutritious complementary foods that provide a variety of nutrients, tastes, and textures, including foods that contain iron [39]. A variety of different foods should be introduced. In this study, on average 6.6% of infants were introduced to solid food before four months, with 93.6% of them before six months. In the Xinjiang Uygur Autonomous Region, in far Western China, a cohort study found that 77.6% of infants were given solid foods by six months [17,23]. The prevalence found here was similar to that of Switzerland, where five percent of infants had received solid food before four months [40], but was higher than the rate in Germany and lower than the rates in Australia and the United States in the early 2000s [41,42,43,44]. 

The median age for the introduction of solid food in Chengdu was five months, similar to a previous study conducted in Germany, but earlier than the median time observed in Beijing (six to eight months) [34]. In this study, the earliest age a solid food was introduced was 1.5 months, whereas in the Xinjiang Uygur Autonomous Region, 5.6% of infants had already been given solid foods in the first two weeks of life [17]. 

Maternal education level was the only factor significantly associated with the introduction of solid food before four months after adjustment for confounding factors. Mothers who had a higher education level (>12 years) were more likely to introduce solid foods to infants before four months than mothers with a lower education level (high school/occupational school or lower degree). Our finding was different from results observed in developed countries, where highly educated mothers tended to delay the introduction of solid food until four months [45,46]. However, it was consistent with a previous study conducted in Beijing [34]. Such apparent association might be related to the current employment situation in China where mothers with higher education levels have more responsibilities from work and often need to return to their employment sooner than other mothers, in order to maintain their position, as their parental leave is generally shorter than their counterparts in Western societies [47]. The early introduction of solid foods has been shown to influence the composition of the gastrointestinal microbiome causing dysbiosis [48]. It is likely that this is linked to long term health outcomes, although research is still needed to quantify the magnitude of the effect and causal pathways [49,50]. 

Though most mothers introduced solid food to infants after four months, the prevalence of introducing solid food before six months was very high. Introduction of solid food after six months can lower morbidity through reducing infections [51]. Almost all public health authorities, including the WHO and the American Academy of Pediatrics, recommend introducing solid foods at or after six months [1,52]. The promotion of appropriate timing for introducing solid food would further improve the health of infants in Chengdu.

There are several limitations that should be considered when interpreting the results of this study. The sample was limited in geographic scope but was chosen to be representative of greater Chengdu, an urban and semi-urban area with a population of 15 million. This population is similar in terms of socio-economic status to other large urban and semi-urban areas of China. The results may not be applicable to more remote rural areas in Sichuan or other provinces of China. There are also regional variations of food culture within China where the food and nutrition practices described in this study may not apply. The recruitment method using random number generation was designed to minimize bias in selection, but this does not mean that the sample can be generalized to a wider population. Major strengths of the study were the frequent interviews which might reduce errors in recall by the mothers, the high response rate, and the low dropout rate during the study. To gain a complete picture of complementary feeding for the entirety China would be very difficult due to the vast area, large population, and many distinct population subgroups. However, Chengdu has many demographic characteristics typical of large Chinese cities and was chosen as a representative location. 

## 5. Conclusions

More than 94% of the infants were given complementary foods (semi-solid or solid foods) before the age of six months and around 10% by four months. More antenatal and postnatal health promotion efforts targeting Chinese mothers are required to highlight the benefits of exclusive breastfeeding until six months and introducing solid foods later than the current practice in Chengdu. Further education about the benefits of delaying the introduction of complementary foods to around six months is also recommended for health professionals including pediatricians, midwives, and community health staff. 

## Figures and Tables

**Table 1:** Demographic information at birth for mothers who introduced solid foods before four months and between four months and six months.

		Introduced before Four Months	Introduction between Four and Six Months	*p* Value
Maternal age (years)	<25	134 (19.4%)	12 (19.0%)	0.943
	≥25	556 (80.6%)	51 (81.0%)	
Maternal education level (years)	<9	82 (11.9%)	10 (15.9%)	0.471
	10–12	145 (21.0%)	10 (15.9%)	
	>12	463 (67.1%)	43 (68.3%)	
Maternal occupation	No formal employment	92 (13.3%)	11 (17.5%)	0.361
	Formal employment	598 (86.7%)	52 (82.5%)	
Family monthly income (RMB)	<5000	150 (23.4%)	18 (30.0%)	0.249
	≥5000	492 (76.6%)	42 (70.0%)	
Mother intention to return to work (months)	≤6	320 (47.0%)	24 (38.7%)	0.211
	>6	361 (53.0%)	38 (61.3%)	
Delivery method	Vaginal	241 (34.9%)	21 (33.3%)	0.799
	Caesarean	449 (65.1%)	42 (66.7%)	
Infant’s gender	Male	360 (52.2%)	32 (50.8%)	0.834
	Female	330 (47.8%)	31 (49.2%)	
Infant’s birth weight (g)	<2500	11 (1.6%)	0 (0.0%)	0.613
	≥2500	679 (98.4%)	53 (100.0%)	
Infant was admitted to NICU	No	671 (97.2%)	62 (98.4%)	0.720
	Yes	19 (2.8%)	1 (1.6%)	
Infant’s first feed with breast milk	No	530 (76.8%)	48 (76.2%)	0.911
	Yes	160 (23.2%)	15 (23.8%)	

NICU = neonatal intensive care unit.

**Table 2:** The rate of introducing solid food before four months by maternal education level.

Maternal Education Level (years)	The Number of Mothers Introducing Solid Foods before Four Months	Percentage Introducing Solid Foods before Four Months	Total Number
<12 years	9	3.6%	250
>12 years	41	8.04%	510
Total	50	6.58%	760

Chi Square 4.286, Degrees of freedom = 1, *p* = 0.0384.

**Table 3:** Types and timing of complementary foods.

Type of Complementary Food	Introduction before Four Months	Introduction between Four and Six Months	Not Introduced by Six Months	Median Age of Introduction (Months)
Rice soup	3 (0.4%)	13 (1.7%)	744 (97.9%)	4.3
Fruit juice	21 (2.8%)	44 (5.8%)	695 (91.4%)	4.0
Vegetable juice	30 (3.9%)	39 (5.1%)	691 (91.0%)	4.0
Rice porridge	0 (0.0%)	42 (5.5%)	718 (94.5%)	5.0
Infant cereal	48 (6.3%)	548 (72.1%)	164 (21.6%)	5.0
Noodles	0 (0.0%)	10 (1.3%)	750 (98.7%)	5.0
Fruit paste	0 (0.0%)	378 (49.7%)	382 (50.3%)	5.0
Vegetable paste	0 (0.0%)	349 (45.9%)	411 (54.1%)	5.0
Protein foods *	5 (0.7%)	397 (52.2%)	358 (47.1%)	5.0
Water	680 (89.5%)	32 (4.2%)	48 (6.3%)	0.8
Chicken liver	0 (0.0%)	7 (0.9%)	753 (99.1%)	5.0

* Solid foods introduced to infants that included egg or meat.

**Table 4:** Factors associated with the introduction of solid food before four months.

Variable	Introduced Solid Food before Four Months				Univariate OR (95% CI)
	No		Yes		
	*n*	%	*n*	%	
Maternal education level					
≤12 years	241	96.4	9	3.6	1
>12 years	469	92.0	41	8.0	2.341 (1.119–4.897) *
Maternal occupation					
No formal employment	103	99.0	1	1.0	1
Formal employment	607	92.5	49	7.5	8.315 **
Maternal residential status in Chengdu					
No	336	95.7	15	4.3	1
Yes	374	91.4	35	8.6	2.096 (1.125–3.907) *
Mother intention to return to work					
>6 months	385	95.5	18	4.5	1
≤6 months	315	90.8	32	9.2	2.173 (1.197–3.945) *

* *p* < 0.05. OR = odds ratio; CI = confidence interval. ** numbers too small to calculate CI

## References

[1] World Health Organisation Appropriate Complementary Feeding. https://www.who.int/elena/titles/complementary_feeding/en/.

[2] Binns C.W., Lee M.K. (2014). Exclusive breastfeeding for six months: The WHO six months recommendation in the Asia Pacific Region. Asia Pac. J. Clin. Nutr..

[3] Binns C., Lee M., Low W.Y. (2016). The Long-Term Public Health Benefits of Breastfeeding. Asia-Pac. J. Public Health.

[4] Rollins N.C., Bhandari N., Hajeebhoy N., Horton S., Lutter C.K., Martines J.C., Piwoz E.G., Richter L.M., Victora C.G., Lancet Breastfeeding Series Group (2016). Why invest, and what it will take to improve breastfeeding practices?. Lancet.

[5] Victora C.G., Bahl R., Barros A.J., Franca G.V., Horton S., Krasevec J., Murch S., Sankar M.J., Walker N., Rollins N.C. (2016). Breastfeeding in the 21st century: Epidemiology, mechanisms, and lifelong effect. Lancet.

[6] Kramer M.S., Kakuma R. (2012). Optimal duration of exclusive breastfeeding. Cochrane Database Syst. Rev..

[7] Pluymen L.P.M., Wijga A.H., Gehring U., Koppelman G.H., Smit H.A., van Rossem L. (2018). Early introduction of complementary foods and childhood overweight in breastfed and formula-fed infants in the Netherlands: The PIAMA birth cohort study. Eur. J. Nutr..

[8] Matvienko-Sikar K., Griffin C., McGrath N., Toomey E., Byrne M., Kelly C., Heary C., Devane D., Kearney P.M. (2019). Developing a core outcome set for childhood obesity prevention: A systematic review. Matern. Child Nutr..

[9] Pearce J., Langley-Evans S.C. (2013). The types of food introduced during complementary feeding and risk of childhood obesity: A systematic review. Int. J. Obes..

[10] World Health Organization Report of the commission on ending childhood obesity. https://www.who.int/end-childhood-obesity/publications/echo-report/en/.

[11] Mihrshahi S., Baur L.A. (2018). What exposures in early life are risk factors for childhood obesity?. J. Paediatr. Child Health.

[12] Oddy W.H. (2017). Breastfeeding, Childhood Asthma, and Allergic Disease. Ann. Nutr. Metab..

[13] Nutrition Section UNICEF Programming Guide: Infant and Young Child Feeding. https://www.ennonline.net/unhcriycfprogrammingguide.

[14] Wieser S., Brunner B., Tzogiou C., Plessow R., Zimmermann M.B., Farebrother J., Soofi S., Bhatti Z., Ahmed I., Bhutta Z.A. (2018). Reducing micronutrient deficiencies in Pakistani children: Are subsidies on fortified complementary foods cost-effective?. Public Health Nutr..

[15] Duong D.V., Binns C.W., Lee A.H. (2005). Introduction of complementary food to infants within the first six months postpartum in rural Vietnam. Acta Paediatr..

[16] Tang L., Lee A.H., Binns C.W. (2015). Predictors of early introduction of complementary feeding: Longitudinal study. Pediatr. Int..

[17] Xu F., Binns C., Lee A., Wang Y., Xu B. (2007). Introduction of complementary foods to infants within the first six months postpartum in Xinjiang, PR China. Asia Pac. J. Clin. Nutr..

[18] Inoue M., Binns C.W. (2014). Introducing solid foods to infants in the Asia Pacific region. Nutrients.

[19] Yu C., Binns C.W., Lee A.H. (2016). Comparison of breastfeeding rates and health outcomes for infants receiving care from hospital outpatient clinic and community health centres in China. J. Child Health Care.

[20] Scott J.A., Aitkin I., Binns C.W., Aroni R.A. (1999). Factors associated with the duration of breastfeeding amongst women in Perth, Australia. Acta Paediatr..

[21] Scott J.A., Binns C.W., Graham K.I., Oddy W.H. (2006). Temporal changes in the determinants of breastfeeding initiation. Birth.

[22] Duong D.V., Binns C.W., Lee A.H. (2004). Breast-feeding initiation and exclusive breast-feeding in rural Vietnam. Public Health Nutr..

[23] Xu F., Binns C., Yu P., Bai Y. (2007). Determinants of breastfeeding initiation in Xinjiang, PR China, 2003-2004. Acta Paediatri..

[24] Qiu L., Zhao Y., Binns C.W., Lee A.H., Xie X. (2009). Initiation of breastfeeding and prevalence of exclusive breastfeeding at hospital discharge in urban, suburban and rural areas of Zhejiang China. Int. Breastfeed J..

[25] WHO Infant and young child feeding: Model chapter for textbooks for medical students and allied health professionals. http://www.who.int/maternal_child_adolescent/documents/9789241597494/en/.

[26] National Health and Medical Research Council Infant Feeding Guidelines for Health Workers. https://www.nhmrc.gov.au/about-us/publications/infant-feeding-guidelines-information-health-workers.

[27] Feng X.L., Xu L., Guo Y., Ronsmans C. (2012). Factors influencing rising caesarean section rates in China between 1988 and 2008. Bull. World Health Organ..

[28] Boerma T., Ronsmans C., Melesse D.Y., Barros A.J.D., Barros F.C., Juan L., Moller A.B., Say L., Hosseinpoor A.R., Yi M. (2018). Global epidemiology of use of and disparities in caesarean sections. Lancet.

[29] Liang J., Mu Y., Li X., Tang W., Wang Y., Liu Z., Huang X., Scherpbier R.W., Guo S., Li M. (2018). Relaxation of the one child policy and trends in caesarean section rates and birth outcomes in China between 2012 and 2016: Observational study of nearly seven million health facility births. BMJ.

[30] Tang L., Qiu L., Yau K., Hui Y., Binns C.W., Lee A.H. (2015). Recent Trends in Gender Ratio at Birth in Hangzhou, China. Vietnam J. Public Health.

[31] Kung’u J.K., Boor K.J., Ame S.M., Ali N.S., Jackson A.E., Stoltzfus R.J. (2009). Bacterial populations in complementary foods and drinking-water in households with children aged 10–15 months in Zanzibar, Tanzania. J. Health Popul. Nutr..

[32] Brown J., Cairncross S., Ensink J.H.J. (2013). Water, sanitation, hygiene and enteric infections in children. Arch. Dis. Child..

[33] Islam M.A., Ahmed T., Faruque A.S.G., Rahman S., Das S.K., Ahmed D., Fattori V., Clarke R., Endtz H.P., Cravioto A. (2012). Microbiological quality of complementary foods and its association with diarrhoeal morbidity and nutritional status of Bangladeshi children. Eur. J. Clin. Nutr..

[34] Li L., Li S., Ali M., Ushijima H. (2003). Feeding practice of infants and their correlates in urban areas of Beijing, China. Pediatr. Int..

[35] Zhang Y., Li H., Xia X. (2008). Breastfeeding practices in nine cities of China and its changes in the past 20 years. Chin. J. Child Care.

[36] Li T., Bindels J.G., Zhang S., Tan Z., Jia N., Liu A., Zhu Z., Dai Y. (2018). A dietary and nutritional status survey among young children in five big cities of China. Asia Pac. J. Clin. Nutr.

[37] World Health Organization The optimal duration of exclusive breastfeeding: Report of an expert consultation. https://www.who.int/nutrition/publications/optimal_duration_of_exc_bfeeding_report_eng.pdf.

[38] National Health and Medical Research Council Infant Feeding Guidelines for Health Workers Evidence Review. https://www.nhmrc.gov.au/about-us/publications/infant-feeding-guidelines-information-health-workers#block-views-block-file-attachments-content-block-1.

[39] Qasem W., Azad M.B., Hossain Z., Azad E., Jorgensen S., Juan S.C.S., Cai C.X., Khafipour E., Beta T., Roberts L.J. (2017). Assessment of complementary feeding of Canadian infants: Effects on microbiome & oxidative stress, a randomized controlled trial. BMC Pediatr..

[40] Dratva J., Merten S., Ackermann-Liebrich U. (2006). The timing of complementary feeding of infants in Switzerland: Compliance with the Swiss and the WHO guidelines. Acta Paediatr..

[41] Rebhan B., Kohlhuber M., Schwegler U., Koletzko B.V., Fromme H. (2009). Infant feeding practices and associated factors through the first 9 months of life in Bavaria, Germany. J. Pediatr. Gastroenterol. Nutr..

[42] Scott J.A., Binns C.W., Graham K.I., Oddy W.H. (2009). Predictors of the early introduction of solid foods in infants: Results of a cohort study. BMC Pediatr..

[43] Fein S.B., Labiner-Wolfe J., Scanlon K.S., Grummer-Strawn L.M. (2008). Selected complementary feeding practices and their association with maternal education. Pediatrics.

[44] Magarey A., Kavian F., Scott J.A., Markow K., Daniels L. (2016). Feeding Mode of Australian Infants in the First 12 Months of Life: An Assessment against National Breastfeeding Indicators. J. Hum. Lact..

[45] Lande B., Andersen L.F., Baerug A., Trygg K.U., Lund-Larsen K., Veierod M.B., Bjorneboe G.E. (2003). Infant feeding practices and associated factors in the first six months of life: The Norwegian infant nutrition survey. Acta Paediatr..

[46] Hendricks K., Briefel R., Novak T., Ziegler P. (2006). Maternal and child characteristics associated with infant and toddler feeding practices. J. Am. Diet. Assoc..

[47] Jia N., Dong X.Y., Song Y.P. (2018). Paid Maternity Leave and Breastfeeding in Urban China. Fem. Econ..

[48] Voreades N., Kozil A., Weir T.L. (2014). Diet and the development of the human intestinal microbiome. Front. Microbiol..

[49] Campoy C., Campos D., Cerdo T., Dieguez E., Garcia-Santos J.A. (2018). Complementary Feeding in Developed Countries: The 3 Ws (When, What, and Why?). Ann. Nutr. Metab..

[50] Isolauri E., Salminen S., Rautava S. (2016). Early Microbe Contact and Obesity Risk: Evidence Of Causality?. J. Pediatr. Gastroenterol. Nutr..

[51] Agostoni C., Przyrembel H. (2013). The timing of introduction of complementary foods and later health. World Rev. Nutr. Diet..

[52] Section on Breastfeeding (2012). Breastfeeding and the use of human milk. Pediatrics.

